# Multi-Omics Elucidates Difference in Accumulation of Bioactive Constituents in Licorice (*Glycyrrhiza uralensis*) under Drought Stress

**DOI:** 10.3390/molecules28207042

**Published:** 2023-10-12

**Authors:** Chengcheng Wang, Dawei Wu, Liying Jiang, Xunhong Liu, Tiantian Xie

**Affiliations:** 1 School of Traditional Chinese Medicine, Jiangsu Vocational College of Medicine, Yancheng 224000, China; wuheyu5@sina.com (D.W.); jly2308@163.com (L.J.); xzmcxtt@126.com (T.X.); 2 School of Pharmacy, Nanjing University of Chinese Medicine, Nanjing 210023, China

**Keywords:** *Glycyrrhiza uralensis*, drought stress, bioactive constituents, multi-omics

## Abstract

Licorice is a frequently applied herb with potential edible and medicinal value based on various flavonoids and triterpenes. However, studies on detailed flavonoid and triterpene metabolism and the molecular basis of their biosynthesis in licorice are very limited, especially under drought conditions. In the present study, we carried out transcriptome, proteome, and metabolome experiments. To ultimately combine three omics for analysis, we performed a bioinformatics comparison, integrating transcriptome data and proteome data through a Cloud platform, along with a simplified biosynthesis of primary flavonoids and triterpenoids in the KEGG pathway based on metabolomic results. The biosynthesis pathways of triterpenes and flavonoids are enriched at both gene and protein levels. Key flavonoid-related genes (*PAL*, *4CL*, *CHS*, *CHI*, *CYP93C*, *HIDH*, *HI4OMT*, and *CYP81E1_7*) and representative proteins (HIDH, CYP81E1_7, CYP93C, and VR) were obtained, which all showed high levels after drought treatment. Notably, one R2R3-MYB transcription factor (Glyur000237s00014382.1), a critical regulator of flavonoid biosynthesis, achieved a significant upregulated expression as well. In the biosynthesis of glycyrrhizin, both gene and protein levels of bAS and CYP88D6 have been found with upregulated expression under drought conditions. Most of the differentially expressed genes (DEGs) and proteins (DEPs) showed similar expression patterns and positively related to metabolic profiles of flavonoid and saponin. We believe that suitable drought stress may contribute to the accumulation of bioactive constituents in licorice, and our research provides an insight into the genetic study and quality breeding in this plant.

## 1. Introduction

*Glycyrrhiza uralensis* Fisch (licorice) is an important medicinal plant for environmental protection. Licorice is rich in the class of triterpenoids and flavonoids, which are responsible for the largest proportion of all chemical compounds [1,2]. Due to these constituents, licorice has been extensively used to treat tremors, respiratory infections, gastritis, and peptic ulcers in traditional Chinese medicine [3]. Recently, many investigations have also recommended that licorice could be developed independently or used in combination to treat COVID-19 [4,5]. Licorice does not only have good performance in medicine but also has important value in food, industry products, and so forth. Our previous research showed that salt stress would increase the content of two major active components of licorice, by increasing the expression of the synthetase gene of skeleton compounds and the downstream glycosyltransferase genes [6,7,8]. It is generally believed that many secondary metabolites of traditional Chinese medicine, i.e., active components, accumulate more under abiotic stress [9,10,11]. In addition, licorice species are capable of tolerating drought conditions [12,13] and, correspondingly, drought stress is also an important factor to promote the biochemical composition of licorice by regulating the synthesis genes [14]. Studying how environmental stress affects the formation and content of active products not only provides a basis for the preparation of environmentally friendly biosynthesis but also has important significance for environmental protection.

At present, with the development of multi-omics technology, the improvement of bioinformatics analysis, and the improvement of molecular biology methods, the research on the biosynthesis of licorice chemicals displays a rapid developing trend. The whole genome and transcriptome of glycyrrhizin have been released [15], and the analysis of the glycyrrhizin biosynthesis pathway has been completed; *CYP88D6* [16], *CYP72A154* [17], *UGT73P12* [18], *GuCSyGT* [19], and *GuUGAT* [20] have greatly promoted the study of licorice saponin synthesis in vitro and also provided a reference for flavonoid biosynthesis. However, the research on the impact of drought stress on the overall change of licorice genes and proteins and the mechanism of the content change of multiple bioactive components remain limited. Multi-omics could describe the changes at different levels from genes to metabolites. According to combination analysis, the key factors in the complete pathway could be identified, leading to the improvement of the quality of cultivated licorice and provision of basic data for the production of bioactive ingredients.

Multi-omics techniques are powerful tools for the identification of quantitative changes in gene and protein expression. Tandem mass tags (TMT), which have been developed for accurate quantification of proteins [21,22] along with transcriptome technology are often applied to compare relative abundances of proteins and genes between the treatment and control groups, especially for those under various environmental stress [23]. In this study, TMTs (tandem mass tags) and reference transcriptome were used for omics analysis of licorice under different drought conditions. Licorice seedlings were subjected to drought stress for 50 days, during which we collected plant height and diameter data of experimental licorice. After 50 days, licorice samples were collected four times to carry out the physiological and biochemical experiments, and metabolite profiles of licorice were determined. According to the comprehensive bioinformatics analysis, we screened primary genes and enzymes that catalyze the synthesis of triterpenoid and flavonoid metabolites. Ultimately, integrated omics data and determination analysis for major bioactive constituents provide a better understanding of the process involved in their differential accumulation under drought stress.

## 2. Results

### 2.1. Morphological Observation and Antioxidant Enzyme Activities of Licorice under Drought Stress

**Figure 1:**
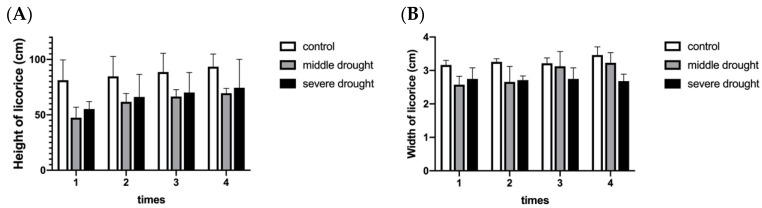
Varieties of licorice height (**A**) and width (**B**) under different drought treatments during 50 d. Each value is a mean ± standard deviation of triplicate assays.

After being stressed for 50 days, the morphological changes of licorice seedlings were clearly observed under different drought stress. In the first place, licorice in all three drought groups displayed a desirable growth trend in terms of plant height, suggesting that licorice had a certain ability to adapt to drought conditions (Figure 1A). However, Figure S1 showed that the leaves of licorice seedlings under severe drought stress are malnourished and sparse, and the plants are relatively short, with a slow growth trend and a diameter of about 2 cm (Figure 1B), indicating that severe drought has affected the biomass of licorice and is not conducive to its sustainable growth. In addition to obvious phenotypic changes, the antioxidant enzyme activity of licorice is also activated under drought environmental conditions, including CAT [24], SOD [25], POD [26], and GR, to eliminate the toxicity of ROS reactive oxygen species [27,28]. As shown in Figure S2, the activities of most antioxidant enzymes showed best performance in the medium drought group, but activities of CAT, SOD, and GR in licorice suffering the severe drought group are even lower than those in the control group, suggesting that severe drought stress has affected the survival of licorice, which is consistent with change of morphological structure. Severe drought stress has also increased the licorice cytotoxicity, and content varieties of these enzymes showed mainly opposite trends between control and drought-treated groups. In general, moderate stress can improve the antioxidant capacity of licorice without affecting normal growth of the plant. Therefore, we selected the moderate drought group and control group for subsequent comparative omics analysis.

### 2.2. GO and KEGG Analysis of DEPs and DEGs

#### 2.2.1. Global Analysis of Protein and Gene Data

The functional annotation of all proteins and genes from Pfam, GO, KEGG, SubCell-Location, and COG are shown in Figure S3. The quality control of identified proteins/genes, matched protein/genes numbers, and other basic information are recorded in our published work [7]. The results suggested that numbers of all kinds of genes were largely higher than proteins, indicating quicker response to drought stress in gene level. Based on normal criteria—namely, FDR < 0.05 and unique peptide numbers, which were set above 2—5760 proteins were obtained from all licorice samples and subjected to comparative analysis. Following the criterion of 95% significance and a 1.2-fold cutoff, a total of 590 proteins were identified as DEPs. As for DEGs, the standards and threshold were the same as before. The quality of data was high, and it could be used for further analysis.

The PCA results showed that control group and drought-stressed groups were clearly separated, and three replicates of each group were clustered, illustrating that these replicates had good repeatability within the group (Figure S4A,C). Among the total 5760 proteins, which were identified and quantified using TMT proteomics, volcano scatter showed that 220 proteins were upregulated, while 370 downregulated (Figure S4B and Table S1) between control group and drought-stressed ones. According to the criteria of DEGs, 25,889, including 806 up- and 486 down-regulated differentially expressed genes (DEGs), were identified in control versus drought-treatment groups (Figure S4D and Table S2). It is worth noting that the number of upregulated DEPs is less than downregulated ones, while the number of regulated DEGs showed opposite trends, indicating differences existing between the protein expression and gene regulation.

#### 2.2.2. Functional Classification and Enrichment Analysis of DEPs and DEGs

A large number of differentially expressed proteins (DEPs) were involved in the cellular component (CC), biological processes (BP), and molecular function (MF) of Gene ontology (GO) terms. Under the category of BP (Figure S5A), metabolic, cellular, and single-organism processes take the top three items. In the MF, many regulated proteins were mainly found in catalytic activity and binding. Apart from these, DEPs, which were associated with the cell, cell parts, organelle, and macromolecular complex, were also observed in the CC category. Interestingly, Figure S5B showed the same GO term results in DEGs. Furthermore, the enrichment analysis suggested that DEPs were involved in various biosynthetic processes, among which more than 20 DEPs were enriched in small molecules, carboxylic acid, and organic acid (Figure S5C). In addition, the kinds of enzyme activity account for a large portion of GO enrichment. With respect to DEGs, Figure S5D showed that catalytic activity was found enriched with the largest number. The second portion is single organism metabolic process, which was consistent with the GO term results of DEPs. Notably, the rich factors of the isoflavonoid biosynthetic and metabolic process displayed significantly higher than others. These findings indicated that drought also causes cell toxicity at the cellular level and stressed that licorice produced different defensive strategies on gene and protein levels to survive harsh circumstances in an efficient way.

To explore which pathways of drought responses DEPs and DEGs participated in, KEGG annotation and enrichment analysis were performed (Figure S6). The result shows that the proteins were mainly found in the metabolism pathways such as amino sugar, energy, and carbohydrate metabolism. The other high, abundant DEPs (Figure S6A) were observed in genetic information processing, indicating that licorice cells are vigorous during the stress-related biological processes. At a genetic level, there exist slight differences. Carbohydrate metabolism was found with above 70 numbers of DEGs (Figure S6B), which ranked second after the signal transduction DEGs of the environmental information processing item. Overall, the metabolism DEGs took the largest portion in all concerned genes. Moreover, enrichment results (Figure S6C) showed that primary and secondary metabolites including the lysine, glycine, serine, triterpenoid, and flavonoid were involved in metabolite biosynthesis. Apart from triterpenoid and flavonoid biosynthesis, the highest rich factor of isoflavonoid biosynthesis in DEGs was obtained (Figure S6D), similar with the findings in GO enrichment. Additionally, plant hormone signal transduction exhibited higher numbers in both DEPs and DEGs, which is notably different from the salt-stressed licorice, suggesting that different stress conditions lead to various tolerant strategies of licorice. Generally, the results of DEPs and DEGs mainly exist in the drought response and metabolic process, especially in DEGs adapting to environmental changes. DEPs and DEGs in the metabolism of the bioactive products concerned have also been observed with differential enrichment, indicating that these metabolites participate in drought stress to varying degrees.

### 2.3. Determination of Multiple Bioactive Constituents

**Figure 2:**
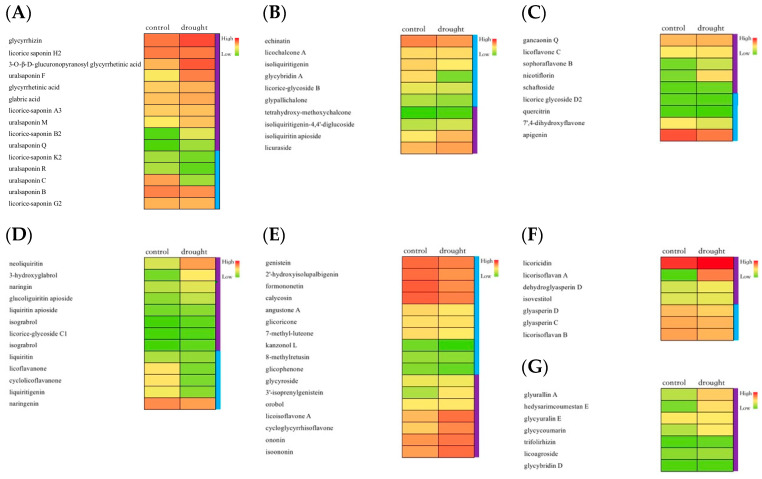
Heat map for metabolite profile for 76 compounds identified in drought group and non-stressed licorice: classes of triterpenoids (**A**), chalcone (**B**), flavonoid (**C**), dihydroflavone (**D**), isoflavonoid (**E**), flavanone (**F**), and others (**G**).

The metabolite profiles of licorice root extracts treated through different water deficit conditions were identified using UFLC-triple TOF-MS/MS, based on our previous published work [29]. A total of 76 metabolites were detectable in both groups and selected with desirable relative contents. The heat map was drawn according to the average value of the relative contents. As in Figure 2, most triterpenoids exhibited higher contents in the drought group than the control, whereas the relative contents of isoflavonoid and chalcone displayed a decreased trend under the stressed condition. Apart from this, other types of flavonoids suggested an up-trend pattern, like flavonoid, dihydroflavone, and flavanone. Additionally, group –C_4_H_8_ showed a high frequency linked to flavonoids, which were in accordance with those findings of our previous work [6]. Furthermore, all other derivatives exhibited increased upregulated contents after drought stress.

### 2.4. Advanced Analysis of Transcriptome and Proteome

#### 2.4.1. Cluster Analysis of Basic Information

**Figure 3:**
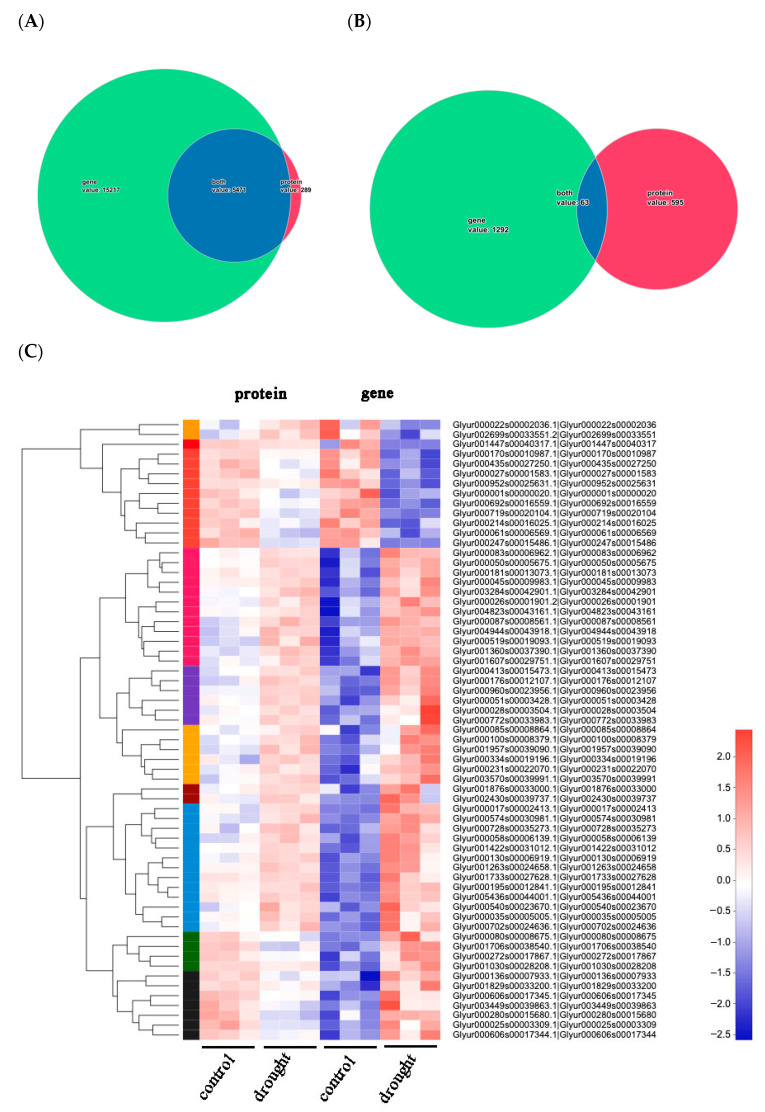
Wayne diagram of relationship between annotated gene/protein (**A**) and DEGs/DEPs (**B**). The green part represents the unique number of genes in the transcriptome project, the red part represents the unique number of proteins in the proteome, and the blue part represents the number of overlaps between proteins and genes that have mapping relationships. Expression patterns of 63 common DEGs and DEPs (**C**). Each column in the figure represents a sample, and each row represents a protein/gene. The colors in the figure represent the relative expression level of the protein/gene in the group of samples. The specific trend of expression level changes can be seen in the numerical annotation under the color bar at the bottom right. On the left is a tree diagram of protein/gene clustering, while on the right is the name of the protein/gene. The closer the two protein/gene branches are, the closer their expression levels are.

We analyzed all the proteins and genes obtained from annotation and differential expression, and the number of gene expressions was much higher than that of protein. Most of the proteins obtained from annotation were products of corresponding genes (Figure 3A,B). Heat maps were generated automatically using the I-Sanger eCloud Platform, which was supported with the R package. In terms of differential expression, there are only 63 common differential expressions between genes and proteins, and the rest are individual differences, revealing that gene expression and protein levels are not completely consistent, which might be a reason of transcription factors involved. Therefore, the combination analysis is more meaningful and convincing. Next, we further analyzed the bioinformatics of 63 common differential expressions. The cluster heat map of related data showed that more than 80% of DEPs and DEGs have the same expression trend (Figure 3B). At the same time, compared with the control group, most DEGs showed an up-regulation pattern, while the level of protein expression was slightly lower than that of genes. Therefore, in the case of external stimulation or long-term stress, the gene expression level is obviously intense, while the functional protein is relatively mild.

#### 2.4.2. Annotation and Enrichment Analysis of GO and KEGG in Common DEPs and DEGs

**Figure 4:**
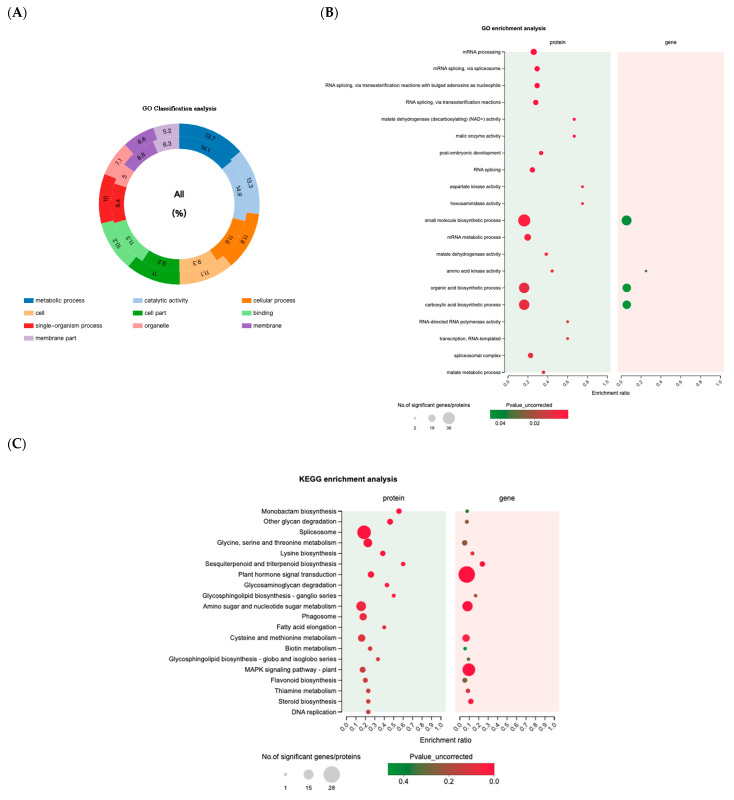
Gene ontology (GO) classification and enrichment of the top 20 terms of DEPs (**A**) and DEGs (**B**). The different colors of pie rings in the figure represent different GO terms, and their area represents the relative proportion of proteins in the GO term. The numbers corresponding to the colors represent the identified proteins and the number of annotated GO terms in the genes. The inner circle represents genes, while the outer circle represents proteins. Kyoto Encyclopedia of Genes and Genomes (KEGG) annotation and enrichment of top 20 pathways of DEPs and DEGs (**C**). The vertical coordinate is GO term. The horizontal axis represents the enrichment rate, and each bubble in the graph represents a GO secondary classification. The size of bubbles is proportional to the number of proteins or genes enriched in this GO secondary classification. The different colors of bubbles represent *p*-value. The top 20 pathways (*p* ≤ 0.05) were used to generate the illustration.

The combined analysis of GO for 63 common differences showed that the highest proportions of the metabolic process and catalytic activity were observed, no matter in gene or protein level, taking more than 13%. Cells and cellular processes have a significant proportion as well, which is consistent with the results of a separate difference analysis, suggesting that licorice cells respond to environmental changes and adapt to drought conditions by activating a series of metabolic and catalytic reactions (Figure 4A). With respect to the functional enrichment analysis, the anabolism of organic acids, carboxylic, and small molecular substances was prominent, illustrating that the drought resistance of licorice promoted the production of these components (Figure 4B). Additionally, the splicing of RNA and transcription process of mRNA were markedly enriched at protein level, which was different from the increase in glycosyltransferase content caused by salt stress, which means that licorice has distinct strategies against two kinds of stresses.

The KEGG enrichment indicated that the expression levels of DEPs and DEGs in primary metabolites, including several amino acids, were similar, and the increase in these amino acids was an important feature of plants to improve their stress resistance in Figure 4C. However, when it comes to the MAPK pathway and plant hormones, the contents of DEPs are remarkably lower than that of DEGs, and the regulation profiles of some genes and proteins are even opposite, indicating that there is intermediate regulation between genes and proteins in the metabolic process of more complex pathways, and the expression patterns of the two are not directly parallel. Regarding the biosynthesis of flavonoids, triterpenes, and other secondary metabolites, the trend of gene and protein expression is consistent, and most of them are upregulated. The findings indicated that drought stress has promoted the biosynthesis of licorice components.

### 2.5. Combined Analysis of Key DEPs and DEGs in Triterpenoid and Flavonoid Pathways

#### 2.5.1. Correlation Analysis among Transcripts, Proteins and Triterpenoid Derivatives

**Figure 5:**
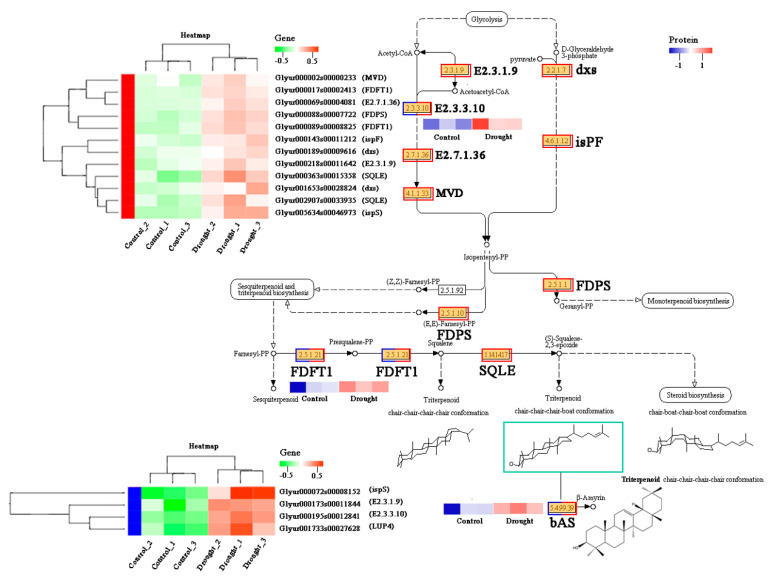
Expression levels of crucial sixteen DEGs and three DEPs involved in triterpenoid biosynthesis. The red and green heat maps represent the differential gene expression on this pathway, while the blue and red heat maps represent the differential protein expression on this pathway.

Triterpenoids and their glycosides are the most important symbolic components of licorice. Almost all triterpenoid compounds of licorice are derived from their boat-type triterpenoid skeleton structure, mainly including glycyrrhizin and glycyrrhetinic acid. In this study, a total of 10 enzymes and 16 genes involved in the steroid biosynthesis pathway were quantified, including 9 up- and 1 downregulated proteins and 16 upregulated genes, respectively (Figure 5 and Figure S8).

To better understand the regulatory network of triterpenoid biosynthesis in licorice, the simplified correlation network among quantitative changes of genes and proteins under different drought stages was clearly illustrated combined with the metabolic profiles of saponins. Based on the result of DEGs and DEPs, which were both enriched in triterpenoid biosynthesis pathway, 16 major key genes were significantly upregulated after drought treatment containing *MVD* (*Glyur000002s00000233*), two *FDFT1* genes (*Glyur000017s00002413* and *Glyur000089s00008825*), *FDPS* (*Glyur000088s00007722*), and *ispF* (*Glyur000143s00011212*), coding corresponding proteins, which catalyze the synthesis of triterpene precursors. At the same time, *LUP4* (*bAS*, *Glyur001733s00027628*), whose counterpart protein acts as a vital rate-limiting enzyme in glycyrrhizin biosynthesis, showed the same markedly increased expression levels in the drought-stressed group. Furthermore, the expression patterns of DEPs on the pathway were shown in Figure 5. Notably, three joint proteins including E2.3.3.10, FDFT1, as well as bAS displayed higher expressions in drought-stressed licorice than untreated ones, leading to a different accumulation of triterpenoid biosynthesis. As expected, determination on the typical triterpenoid content, namely, glycyrrhizin acid and glycyrrhetinic acid, in different groups showed that both of them accumulated less in the control group. Contents of most downstream licorice saponins like uralsaponin F, M, Q as well as licorice saponin H2 and B2 (Figure 2A) are in good agreement with the corresponding multi-omics values.

#### 2.5.2. Correlation Analysis among Transcripts, Proteins, and Flavonoid Derivatives

Flavonoids along with their derivatives account for the largest proportion of the total components of licorice [30]. Flavonoids are synthesized from phenylpropanoids, which existed in these significantly enriched pathways [31,32,33]. The DEGs and DEPs associated with these two pathways were identified based on the enriched KEGG pathways, consisting of 21 upregulated genes, such as *4CL* (*Glyur000051s00003417*), *PAL* (*Glyur007164s00047115*), *C3′H* (*Glyur000013s00003277*), *CHS* (*Glyur000051s00003428*), *FLS* (*Glyur002747s00042594*), and one downregulated *E1.11.1.7* (*Glyur001544s00035568*).

**Figure 6:**
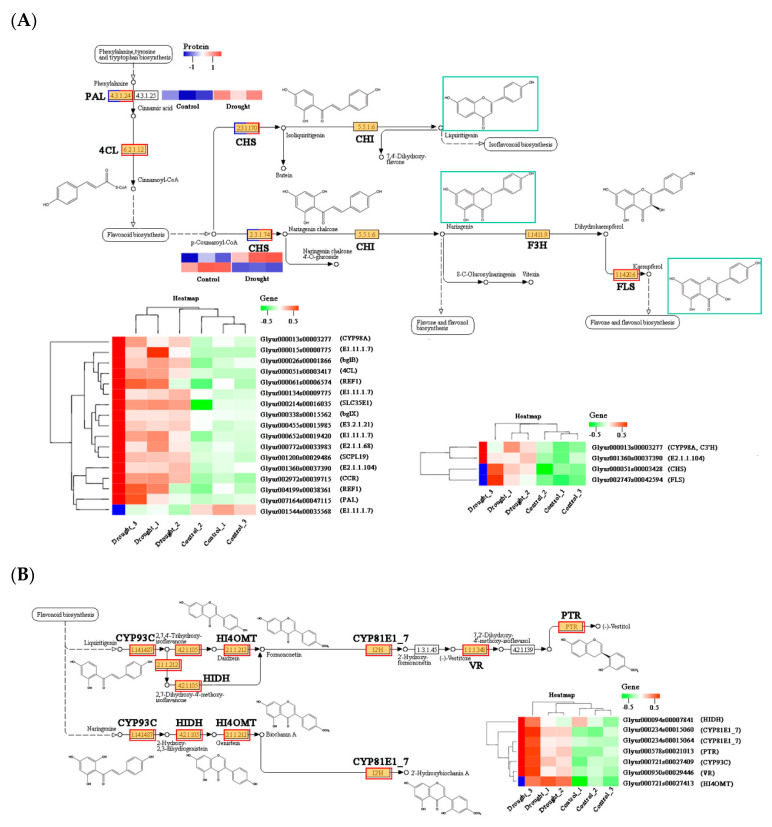
Expression levels of crucial twenty-eight DEGs and three DEPs involved in flavonoid derivatives (**A**) and isoflavonoid biosynthesis (**B**). The red and green heat maps represent the differential gene expression on this pathway, while the blue and red heat maps represent the differential protein expression on this pathway.

According to the joint flavonoid types in licorice and crucial enzymes related to synthesis (Figures S9 and S10), the pathway was then simplified, and interaction networks were organized in Figure 6. As a result, PAL and one CHS, playing the rate-limiting role in regulating flavonoid biosynthesis, displayed higher expressions in drought-stressed licorice, while the other CHS showed the opposite trend. Despite the higher expression of the FLS gene in the drought-stressed group, which participates in the first step of flavone and flavonol synthesis, final products in this network, especially liquiritigenin and naringenin, were still found with lower levels in the stressed group (Figure 2D). Additionally, liquiritigenin is one of the starting compounds in the isoflavonoid biosynthesis. A total of seven significant DEGs in isoflavonoid biosynthesis, including *HIDH* (*Glyur000094s00007841*), CYP81E1_7 (*Glyur000234s00015060*, *Glyur000234s00015064*), *PTR* (*Glyur000578s00021013*), *CYP93C* (*Glyur000721s00027409*), *VR* (*Glyur000950s00029446*), and *HI4OMT* (*Glyur000721s00027413*) exhibited remarkably upregulated patterns through drought treatment. However, no DEP was enriched on the isoflavonoid biosynthesis pathway, illustrating the regulation of differences between genes and proteins. While metabolic results showed that contents of main downstream flavonoid derivatives were with different profiles due to their complicated structures. In detail, isoflavonoids and chalcones such as formononetin and isoliquiritigenin were found with lower contents (Figure 2C,D) after drought treatment, while most flavonoid apiosides, flavanes, and final products such as liquiritin apioside, licorisoflavan A, and licoagroside were observed with higher contents in treated licorice (Figure 2E–G). To study the precise effects of water-deficient treatment on the accumulation of flavonoids in licorice roots, specific DEGs or DEPs should be explored further.

### 2.6. Quantitative Analysis of Selected Genes and Proteins Involved in Flavonoid and Triterpenoid Biosynthesis

The expression profiles of crucial DEGs and DEPs involved in flavonoid and triterpenoid biosynthesis were confirmed with qRT-PCR and PRM validation. Combined with the pre-detected results of DEGs and DEPs mentioned above in licorice, we ultimately selected seven genes consisting of *bAS*, *PAL*, *CHS*, *HI4OMT*, *HIDH*, *CYP81E_7*, and CY*P93C* and seven proteins including bAS, PAL, CHS, FDFT1, E2.3.3.10, VR, and CYP93C. Among which, bAS, PAL, and CHS are key DEGs and DEPs associated with triterpenoid and flavonoid biosynthesis, respectively. Different from DEGs, two DEPs were annotated as CHS, and they showed different expression patterns. Basically, FDFT1 in DEPs showed the same trend of increased expression in the pathway of triterpenoid biosynthesis, but there was no difference in the expression levels of E2.3.3.10. The rest are joint DEGs and DEPs involved in the biosynthesis of isoflavone. VR and CYP93C were not DEPs, but we chose them as counterparts of DEGs, the findings of which demonstrated that both of them showed no significant expressed levels between drought-stressed and control groups. Whereas DEGs (*HI4OMT*, *HIDH*, *CYP81E_7*, and CY*P93C*) were remarkably upregulated in drought group, being highly consistent with the expression patterns in transcriptome data.

## 3. Discussion

The phytochemicals of licorice investigated in the present study have been applied to various industrial and medicinal utilities. While the resources of licorice have dramatically declined, the demands on bioactive compounds—glycyrrhizin, for example—in the market is fast growing [34]. In our previous study, we used integrated approaches to identify many important enzymes and related genes, revealing the complex processes involved in regulating metabolite biosynthesis in licorice under different salt treatments. Although the increasing evidence showed that salinity and drought would dramatically influence the accumulation of licorice metabolites [35], the molecular mechanisms regarding the biosynthesis of these constituents associated with water deficiency have not been clarified, which seriously hinder the breeding of high-quality licorice and the protection of existing sources.

In the present study, we conducted drought-stressed experiments to study differences in metabolites from proteomic and genetic levels. At first, the different contents of H_2_O_2_-scavenging proteins, such as SOD, were observed, and biomass changes also occurred for 50 days. These results were consistent with previous reports, which means licorice has enhanced its tolerance confronting the water shortage.

**Figure 7:**
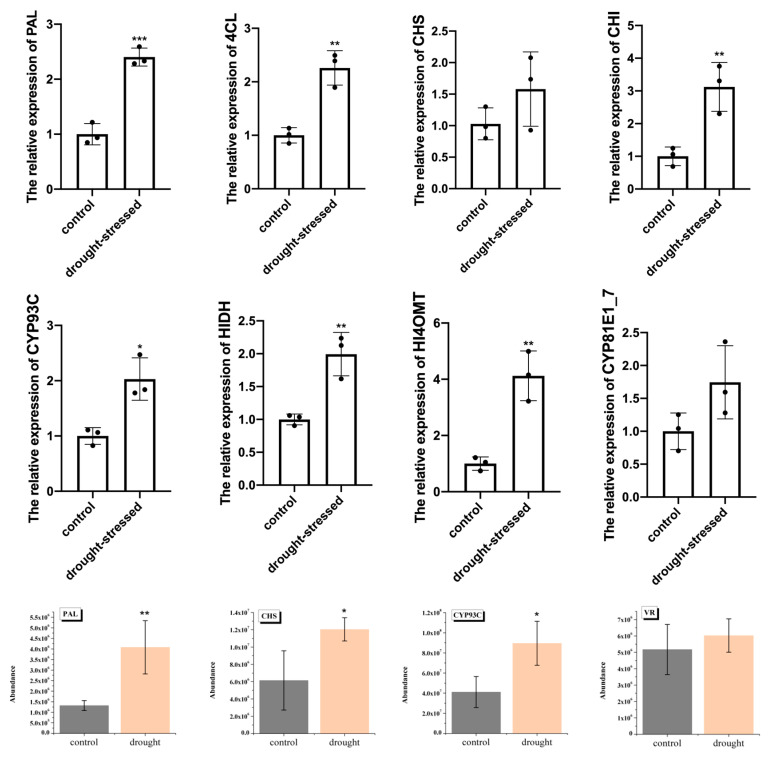
Expression patterns of eight flavonoid-related genes (PAL, 4CL, CHS, CHI, CYP93C, HIDH, HI4OMT, and CYP81E1_7) and four proteins (PAL, CHS, CYP93C, and VR). Data represent means of three biological replicates. Error bars indicate ± SD. Asterisks (*) show significant differences between licorice-treated ones and those not treated with selenium (* *p* < 0.05; ** *p* < 0.01; *** *p* < 0.001).

Flavonoids contribute to considerable bioactive secondary metabolites of medicinal plants and crops [36,37,38]. The chemical scaffolds of flavonoids lead its specific properties such as typical ROS scavenging [39], especially functions under stress conditions. Accumulating research proved that flavonoid production under drought stress would significantly increase among different groups [40,41,42,43]. Correspondingly, various environmental stress would alter the expression of flavonoid biosynthetic proteins and genes. Flavonoid biosynthesis starts from phenylpropanoids consisting of early pathway enzymes, like PAL and 4CL, as well as the late pathway enzymes, such as CHS, CHI, F3H, FLS, LAR, and others [44,45]. Figure 7 showed that identified DEGs enriched on the biosynthesis pathway of flavonoids were upregulated from chemical scaffolds to subsequent modifications. Despite less DEPs found in flavonoids synthesis, these proteins associated with corresponding genes, like HIDH, CYP81E1_7, CYP93C, and VR, all showed similar higher expressed trends, which is in accordance with accumulation pattern of related flavonoids (Figure 7). The results suggest that licorice seedlings enhance drought tolerance based on increased flavonoid metabolic activities and increased contents of superoxide-scavenging enzymes.

Many transcription factors (TFs) have been proven to resist abiotic stress. Overexpression of MYB increases oxidative activity and enhances drought tolerance in Arabidopsis via overaccumulation of flavonoids [46]. In the present study, differentially expressed TFs were also obtained, mainly containing two important families of bHLH and MYB (Figure S7 and Table S3). These results are in accordance with our previous studies on salt-stressed licorice. Specifically, one member of R2R3-MYB TFs (Glyur000237s00014382.1), a homologous gene of AtMYB12, was reported to be closely associated with synthesis of flavonols and flavanones [47] such as kaempferol, dihydromyricetin, neohesperidin, and naringin. In this work, the Log2FC (drought/control) of Glyur000237s00014382.1 > 3, achieving a significant upregulated expression as well as a similar pattern to primary proteins including PAL, one CHS, and all crucial genes on the biosynthesis pathways of flavonoid and derivatives. These findings also suggested that Glyur000237s00014382.1 could be a critical regulator of flavonoid biosynthesis in licorice under drought stress.

**Figure 8:**
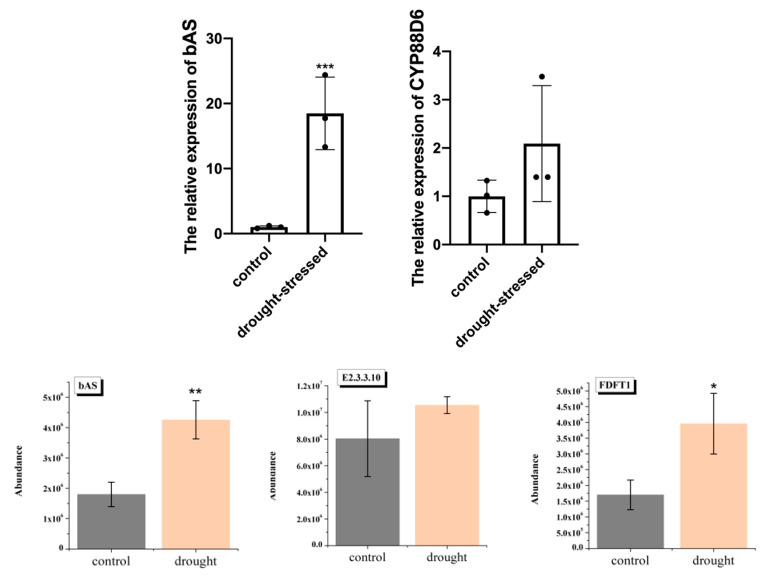
Expression patterns of two triterpenoid-related genes (bAS and CYP88D6) and three proteins (bAS, E2.3.3.10, and FDFT1). Data represent means of three biological replicates. Error bars indicate ± SD. Asterisks (*) show significant differences between licorice-treated ones and those not treated with selenium (* *p* < 0.05; ** *p* < 0.01; *** *p* < 0.001).

In terms of the triterpenoid saponins, predominant types of licorice derived from glycyrrhizin, the classic oleanane structure, stemming from the mevalonic acid pathway of terpene backbone. Several investigations have suggested that triterpenoid saponins have been conducive to the adaptation of plants in water-deficit conditions [48,49,50]. Currently, the pathway of glycyrrhizin has been completely clarified, obtaining bAS, two key CYP450 enzymes (CYP88D6 and CYP72A154), and two UDPs (GuCSyGT and UGT73P12). Our study identified upregulated bAS in the drought-stressed group both on gene and protein levels. Besides, *CYP88D6* showed a significantly higher expression on stressed groups according to qRT-PCR (Figure 8). Apart from this, higher expressed patterns of *E2.3.3.10*, *FDFT1*, and other upstream genes of glycyrrhizin have been observed under drought conditions, which were positively related to the contents of triterpenoid saponins. In turn, the bioactivity of these compounds is responsible for providing defensive strategies for the processes occurring water shortage [51,52]. Additionally, the correlation analysis among multi-omics and metabolite profiling implied that it is an efficient method for identifying influential factors that are involved in biosynthesis of concerned constituents.

## 4. Materials and Methods

### 4.1. Plant Materials and Drought Treatments

The licorice cultivars were obtained and planted referring to the description of our previous work [6,8]. Experimental licorice seedlings grow naturally until they were alive and germinated. Three levels of drought treatments were then designed as follows: normal watering (control group, 60–70% water), moderate drought (40–50% water), and severe drought (30–40% water). The whole period of drought stress lasted 50 days. Ultimately, licorice roots were harvested, cleaned with phosphate-buffered saline (PBS), and immediately frozen −80 °C, using liquid nitrogen for subsequent experiments and multi-omics analysis. The rest were collected as voucher specimens.

### 4.2. Physiological Experiment

Fresh leaves of licorice under different drought treatments were collected and weighed. The assays, including superoxide dismutase (SOD), peroxidase (POD), catalase (CAT), and glutathione reductase (GR) activities, were carried out as described before. All experiments were conducted using assay kits offered by the Nanjing Jiancheng Bioengineering Institute (Nanjing, China). Each reaction solution (200 μL) was detected under a UV-visible absorption using a multimode microplate reader (SpectraMax M5, San Jose, CA, USA).

### 4.3. Proteome Experiment

#### 4.3.1. Protein Extraction, Digestion, and TMT Labeling

Protein extraction, digestion, and TMT labeling were performed according to our published protocols with minor modification. Briefly, three independent biological replicates of licorice roots were grounded and homogenized in a shaker tube containing 1% polyvinylpolypyrrolidone (PVPP) and the right amount of borax/PVPP/phenol (BPP) solution. The supernatant was centrifugated and shaken (4 °C, 12,000× *g*, 20 min) with an equal volume of Tris-saturated phenol. Then, the phenol phase was collected and re-extracted to precipitate protein by adding cold ammonium sulfate-saturated methanol and incubating at −20 °C overnight. The obtained proteins were washed and dissolved in lysis buffer. Total protein concentration measured using the BCA method [7].

Protein digestion protocol was based on our previous work. All collected proteins were transferred to a new tube followed by trypsin digestion with a substrate ratio of 1:50 (*w*/*w*) overnight (maintained at 37 °C). The digested peptides for labeling were labeled with a TMT reagent kit according to the manufacturer’s instructions (Thermo Fisher Scientific, Waltham, MA, USA). After tagging, the TMT-labeled samples were pooled and analyzed with a Thermo Scientific Vanquish F UHPLC system connected to a Q-Exactive hybrid quadrupole Orbitrap mass spectrometer (Thermo Fisher Scientific, Waltham, MA, USA).

#### 4.3.2. High-pH Separation and LC–MS/MS Analysis

The TMT-tagged peptides were mixed and graded as described before. A total of 30 fractions were collected and combined into 15 fractions. Each of them was dried via vacuum centrifugation. The peptides were dissolved, and the supernatant was transferred to the sample tube. The nanoLC–MS/MS was carried out using a Q-Exactive MS (Thermo Scientific) coupled online to the UPLC system (Thermo Dionex). The peptide was loaded onto a C18 column (75 μm × 25 cm, Thermo, Waltham, MA, USA) at a flow rate of 300 nL/min. A linear gradient referred to our published work. The most intense signals were selected for higher-energy collisional dissociation (HCD) fragmentation from the survey scan (*m*/*z* 300–1800). HCD spectra in the Orbitrap were set to 17,500 at *m*/*z* 200, and the fixed first mass was set to *m*/*z* 100.

#### 4.3.3. Parallel Reaction Monitoring Analysis

Abundance levels of 11 detectable DEPs, each of which includes three biological replicates, associated with metabolism of flavonoid and triterpenes were selected from the TMT data. A parallel reaction monitoring (PRM) was performed to confirm obtained proteins by quantifying the expression. Signature peptides for the target proteins were defined, and only unique peptide sequences were selected for PRM analysis on a Q Exactive Plus mass spectrometer (Thermo Scientific). The liquid chromatography parameters, scan range, electrospray voltage, and Orbitrap resolution were the same as those for the TMT methods in present study [53].

### 4.4. Transcriptome Experiment

#### 4.4.1. RNA Isolation and cDNA Library Construction

The total RNA of licorice roots was extracted from three individual biological replicates of frozen licorice root using the Trizol Total RNA Isolation Kit (Sangon Biotech, Shanghai, China) according to the manufacturer’s instructions. The purified RNA requires a concentration ≥ 50 ng/μL, and the OD260/280 value should be based on a ratio of 1.8–2.0. mRNA can be reverse-transcribed from total RNA for analysis of transcriptome information through A-T base pairing with Oligo (dT) magnetic beads and ployA (Invitrogen, Carlsbad, CA, USA). The cDNA library construction for the Illumina sequencing was carried out as detailed.

#### 4.4.2. Transcriptome Sequencing and Functional Annotation

The raw data were firstly filtered to remove reads with low-quality and contaminated adaptors. The remaining high-quality data were mapped to the reference genome sequence of *Glycyrrhiza uralensis* (https://ddbj.nig.ac.jp/resource/bioproject/PRJDB3943) to acquire the unigenes using HISAT2 software (http://ccb.jhu.edu/software/hisat2/index.shtml) according to standard procedure. All assembled unigenes were subjected to a basic local alignment search tool BLASTX (http://blast.ncbi.nlm.nih.gov/Blast.cgi) in the non-redundant NCBI protein database (Nr), Swiss-Prot and clusters of orthologous groups. KOBAS were applied for the Gene ontology function annotation and Kyoto Encyclopedia of Genes and Genomes for studying related metabolic pathways, respectively.

#### 4.4.3. Quantitative Real-Time Polymerase Chain Reaction Analysis

To validate our transcriptome data, qRT-PCR was conducted to quantify a mRNA expression profile of the 11 genes (PAL, 4CL, CHS, CHI, CYP93C, HIDH, HI4OMT, CYP72A154, CYP88D6, and bAS), coding for representative enzymes in flavonoids and triterpene biosynthesis. Extraction of total RNA was according to the manufacturer’s instructions of Trizol Total RNA Isolation Kit (Sangon Biotech, Shanghai, China). Primer 3.0 software was employed to design primers. The PCR procedure and calculation of gene levels referred to the standard.

### 4.5. Metabolite Profiles of Licorice Bioactive Constituents

Sample preparation of licorice roots and analysis assays were performed based on previous studies with minor modifications [29]. The ultrafast liquid chromatography (UFLC)–triple time-of-flight tandem mass spectrometry (TOF-MS/MS) was employed, which was equipped with an ESI source to acquire the MS data. The Analyst TF 1.6 software and PeakView1.2 software were used to collect data in the negative ion mode and to identify the potential chemicals in the control and drought-stressed licorice samples, respectively. Other experimental conditions were as described before.

### 4.6. Data Processing

The I-Sanger eCloud Platform, established by the Majorbio Biopharm Technology Co., Ltd. (Shanghai, China), was used to analyze the proteomic and transcriptomic data, as well as the co-analysis regarding these two omics experiments. Graphs were charted using Origin Pro 8 software. The mean values and standard deviation were calculated with the measurements of the three replicates. All experimental data were statistically analyzed using SPSS 16, and the significance threshold was set as *p* < 0.05.

## 5. Conclusions

Understanding of the biosynthetic pathway would advance the breeding licorice cultivars as functional food or herbal medicine of high quality with enhanced, effective compounds. Furthermore, some of these DEGs and proteins involved in the biosynthesis pathways were also validated. Our research provides an insight into the role of flavonoid and saponin biosynthetic pathways in drought tolerance and potential markers for the further engineering of a higher production of these beneficial compounds in licorice for industrial and medicinal uses.

## Data Availability

No new data were created.

## Supplementary Materials

The following supporting information can be downloaded at https://www.mdpi.com/article/10.3390/molecules28207042/s1, Figure S1: morphology and phenotype profiles of four licorice seedling groups after 50 days under different water deficit conditions; Figure S2: dynamic contents of antioxidases POD (A), SOD (B), CAT (C), and GR (D) in all licorice groups of different treatments; Figure S3: the functional annotation of all proteins (A) and genes (B) from Pfam, GO, KEGG, SubCell-Location, and COG; Figure S4: Principal component analysis (PCA) of DEPs (A) and DEGs (C); volcano map analysis of DEPs (B) and DEGs (D) (red, green, and gray points represent upregulated, downregulated, and unchanged genes, respectively); Figure S5: Gene ontology (GO, cellular component, biological process, and molecular function) classification of top 20 terms of DEPs (A) and DEGs (B); GO enrichment of top 20 terms of DEPs (C) and DEGs (D); the top 20 pathways (*p* ≤ 0.05) were used to generate the illustration; Figure S6: Kyoto Encyclopedia of Genes and Genomes (KEGG) annotation of top 20 pathways of DEPs (A) and genes (B); KEGG enrichment of top 20 pathways of DEPs (C) and DEGs (D); the top 20 pathways (*p* ≤ 0.05) were used to generate the illustration; Figure S7: transcription factor prediction of DEGs in licorice; Figure S8: metabolic pathway maps of differentially expressed proteins involved in sesquiterpenoid and triterpenoid biosynthesis in KEGG enrichment (green for DEPs); Figure S9: metabolic pathway maps of differentially expressed proteins involved in phenylpropanoid biosynthesis in KEGG enrichment (green for DEPs); Figure S10: metabolic pathway maps of differentially expressed proteins involved in flavonoid biosynthesis in KEGG enrichment (Green for DEPs). Table S1: detailed information of DEPs based on different drought treatments; Table S2: detailed information of DEGs based on different drought treatments; Table S3: differentially expressed transcription factors.

## References

[1] Xiang C., Qiao X., Ye M., Guo D.A. (2012). Classification and distribution analysis of components in Glycyrrhiza using licorice compounds database. Acta Pharm. Sin..

[2] Song W., Si L., Ji S., Wang H., Fang X.M., Yu L.Y., Li R.Y., Liang L.N., Zhou D., Ye M. (2014). Uralsaponins M-Y, antiviral triterpenoid saponins from the roots of Glycyrrhiza uralensis. J. Nat. Prod..

[3] Wang X., Zhang H., Chen L., Shan L., Fan G., Gao X. (2013). Liquorice, aunique “guide drug” of traditional Chinese medicine: A review of its role in drug interactions. J. Ethnopharmacol..

[4] Bailly C., Vergote G. (2020). Glycyrrhizin: An alternative drug for the treatment of COVID-19 infection and the associated respiratory syndrome?. Pharmacol. Ther..

[5] Sikander M., Malik S., Rodriguez A., Yallapu M.M., Narula A.S., Satapathy S.K., Dhevan V., Chauhan S.C., Jaggi M. (2020). Role of Nutraceuticals in COVID-19 Mediated Liver Dysfunction. Molecules.

[6] Wang C.C., Chen L.H., Cai Z.C., Chen C.H., Liu Z.X., Liu S.J., Zou L.S., Tan M.X., Chen J.L., Liu X.H. (2021). Metabolite Profiling and Transcriptome Analysis Explains Difference in Accumulation of Bioactive Constituents in Licorice (Glycyrrhiza uralensis) Under Salt Stress. Front. Plant Sci..

[7] Wang C.C., Chen L.H., Cai Z.C., Chen C.H., Liu Z.X., Liu X.H., Zou L.S., Chen J.L., Tan M.X., Wei L.F. (2020). Comparative Proteomic Analysis Reveals the Molecular Mechanisms Underlying the Accumulation Difference of Bioactive Constituents in Glycyrrhiza uralensis Fisch under Salt Stress. J. Agric. Food. Chem..

[8] Wang C.C., Chen L.H., Cai Z.C., Chen C.H., Liu Z.X., Liu X.H., Zou L.S., Chen J.L., Tan M.X., Wei L.F. (2020). Dynamic Variations in Multiple Bioactive Constituents under Salt Stress Provide Insight into Quality Formation of Licorice. Molecules.

[9] Wang F., Xu Z., Fan X., Zhou Q., Cao J., Ji G., Jing S., Feng B., Wang T. (2019). Transcriptome Analysis Reveals Complex Molecular Mechanisms Underlying UV Tolerance of Wheat (*Triticum aestivum* L.). J. Agric. Food Chem..

[10] Zhou Y., Yao L., Huang X., Li Y., Wang C., Huang Q., Yu L., Pan C. (2023). Transcriptomics and metabolomics association analysis revealed the responses of Gynostemma pentaphyllum to cadmium. Front. Plant Sci..

[11] Yu F., Wang Q., Wei S., Wang D., Fang Y., Liu F., Zhao Z., Hou J., Wang W. (2015). Effect of genotype and environment on five bioactive constituents of cultivated licorice (*Glycyrrhiza uralensis*) populations in northern China. Biol. Pharm. Bull..

[12] Hosseini M.S., Samsampour D., Ebrahimi M., Abadía J., Khanahmadi M. (2018). Effect of drought stress on growth parameters, osmolyte contents, antioxidant enzymes and glycyrrhizin synthesis in licorice (*Glycyrrhiza glabra* L.) grown in the field. Phytochemistry.

[13] Li Y., Jiang D., Liu X.Y., Li M., Tang Y.F., Mi J., Ren G.X., Liu C.S. (2023). Multi-Omics Analysis Provides Crucial Insights into the Drought Adaptation of Glycyrrhiza uralensis Fisch. J. Agric. Food Chem..

[14] Nasrollahi V., Mirzaie-Asl A., Piri K., Nazeri S., Mehrabi R. (2014). The effect of drought stress on expression of key genes involved in biosynthesis of triterpenoid saponins in licorice (*Glycyrrhiza glabra*). Phytochemistry.

[15] Mochida K., Sakurai T., Seki H., Yoshida T., Takahagi K., Sawai S., Uchiyama H., Muranaka T., Saito K. (2017). Draft genome assembly and annotation of Glycyrrhiza uralensis, a medicinal legume. Plant J..

[16] Seki H., Ohyama K., Sawai S., Mizutani M., Ohnishi T., Sudo H., Akashi T., Aoki T., Saito K., Muranaka T. (2008). Licorice beta-amyrin 11-oxidase, a cytochrome P450 with a key role in the biosynthesis of the triterpene sweetener glycyrrhizin. Proc. Natl. Acad. Sci. USA.

[17] Seki H., Sawai S., Ohyama K., Mizutani M., Ohnishi T., Sudo H., Fukushima E.O., Akashi T., Aoki T., Saito K. (2011). Triterpene Functional Genomics in Licorice for Identification of CYP72A154 Involved in the Biosynthesis of Glycyrrhizin. Plant Cell.

[18] Nomura Y., Seki H., Suzuki T., Ohyama K., Mizutani M., Kaku T., Tamura K., Ono E., Horikawa M., Sudo H. (2019). Functional specialization of UDP-glycosyltransferase 73P12 in licorice to produce a sweet triterpenoid saponin, glycyrrhizin. Plant J..

[19] Chung S.Y., Seki H., Fujisawa Y., Shimoda Y., Hiraga S., Nomura Y., Saito K., Ishimoto M., Muranaka T. (2020). A cellulose synthase-derived enzyme catalyses 3-O-glucuronosylation in saponin biosynthesis. Nat. Commun..

[20] Xu G.J., Cai W., Gao W., Liu C.S. (2016). A novel glucuronosyl transferase has an unprecedented ability to catalyse continuous two step glucuronosylation of glycyrrhetinic acid to yield glycyrrhizin. New Phytol..

[21] Zhu W., Zhang Y., Ren C.H., Cheng X., Chen J.H., Ge Z.Y., Sun Z.P., Zhuo X., Sun F.F., Chen Y.L. (2020). Identification of proteomic markers for ram spermatozoa motility using a tandem mass tag (TMT) approach. J. Proteom..

[22] Du W., Xiong C.W., Ding J., Nybom H., Ruan C.J., Guo H. (2019). Tandem Mass Tag Based Quantitative Proteomics of Developing Sea Buckthorn Berries Reveals Candidate Proteins Related to Lipid Metabolism. J. Proteome Res..

[23] Zhang C., Yao X., Ren H., Chang J., Wang K. (2019). RNA-Seq Reveals Flavonoid Biosynthesis-Related Genes in Pecan (*Carya illinoinensis*) Kernels. J. Agric. Food Chem..

[24] Baker A., Lin C.C., Lett C., Karpinska B., Wright M.H., Foyer C.H. (2023). Catalase: A critical node in the regulation of cell fate. Free Radic. Biol. Med..

[25] Saed-Moucheshi A., Sohrabi F., Fasihfar E., Baniasadi F., Riasat M., Mozafari A.A. (2021). Superoxide dismutase (SOD) as a selection criterion for triticale grain yield under drought stress: A comprehensive study on genomics and expression profiling, bioinformatics, heritability, and phenotypic variability. BMC Plant Biol..

[26] Li L., Yi H. (2022). Enhancement of drought tolerance in Arabidopsis plants induced by sulfur dioxide. Ecotoxicology.

[27] Hasanuzzaman M., Parvin K., Bardhan K., Nahar K., Anee T.I., Masud A.A.C., Fotopoulos V. (2021). Biostimulants for the Regulation of Reactive Oxygen Species Metabolism in Plants under Abiotic Stress. Cells.

[28] Mittler R., Zandalinas S.I., Fichman Y., Van Breusegem F. (2022). Reactive oxygen species signalling in plant stress responses. Nat. Rev. Mol. Cell Biol..

[29] Wang C.C., Cai Z.C., Shi J.J., Chen S.Y., Tan M.X., Chen J.L., Chen L.H., Zou L.S., Chen C.H., Liu Z.X. (2019). Comparative Metabolite Profiling of Wild and Cultivated Licorice Based on Ultra-Fast Liquid Chromatography Coupled with Triple Quadrupole-Time of Flight Tandem Mass Spectrometry. Chem. Pharm. Bull..

[30] Xing G.X., Li N., Wang T., Yang M.Y. (2003). Research progress of flavonoids in liquorice. China J. Chin. Materia Medica.

[31] Wu C., Wang Y., Sun H. (2023). Targeted and untargeted metabolomics reveals deep analysis of drought stress responses in needles and roots of Pinus taeda seedlings. Front. Plant Sci..

[32] Karlson CK S., Mohd Noor S.N., Khalid N., Tan B.C. (2022). CRISPRi-Mediated Down-Regulation of the Cinnamate-4-Hydroxylase (C4H) Gene Enhances the Flavonoid Biosynthesis in Nicotiana tabacum. Biology.

[33] Liu W., Feng Y., Yu S., Fan Z., Li X., Li J., Yin H. (2021). The Flavonoid Biosynthesis Network in Plants. Int. J. Mol. Sci..

[34] Wang C.C., Chen L.H., Xu C.Q., Shi J.J., Chen S.Y., Tan M.X., Chen J.L., Zou L.S., Chen C.H., Liu Z.X. (2020). A Comprehensive Review for Phytochemical, Pharmacological, and Biosynthesis Studies on Glycyrrhiza spp.. Am. J. Chin. Med..

[35] Zhang W., Xie Z., Wang L., Li M., Lang D., Zhang X. (2017). Silicon alleviates salt and drought stress of Glycyrrhiza uralensis seedling by altering antioxidant metabolism and osmotic adjustment. J. Plant Res..

[36] Li H., Lv Q., Ma C., Qu J., Cai F., Deng J., Huang J., Ran P., Shi T., Chen Q. (2019). Metabolite Profiling and Transcriptome Analyses Provide Insights into the Flavonoid Biosynthesis in the Developing Seed of Tartary Buckwheat (*Fagopyrum tataricum*). J. Agric. Food Chem..

[37] Liu S., Zhang H., Yuan Y. (2022). A Comparison of the Flavonoid Biosynthesis Mechanisms of Dendrobium Species by Analyzing the Transcriptome and Metabolome. Int. J. Mol. Sci..

[38] Dong X., Chen W., Wang W., Zhang H., Liu X., Luo J. (2014). Comprehensive profiling and natural variation of flavonoids in rice. J. Integr. Plant Biol..

[39] Agati G., Brunetti C., Di Ferdinando M., Ferrini F., Pollastri S., Tattini M. (2013). Functional roles of flavonoids in photoprotection: New evidence, lessons from the past. Plant Physiol. Biochem..

[40] Kubra G., Khan M., Munir F., Gul A., Shah T., Hussain A., Caparrós-Ruiz D., Amir R. (2021). Expression Characterization of Flavonoid Biosynthetic Pathway Genes and Transcription Factors in Peanut Under Water Deficit Conditions. Front. Plant Sci..

[41] Kleinwächter M., Selmar D. (2015). New insights explain that drought stress enhances the quality of spice and medicinal plants: Potential applications. Agron. Sustain. Dev..

[42] Gharibi S., Sayed Tabatabaei B.E., Saeidi G., Talebi M., Matkowski A. (2019). The effect of drought stress on polyphenolic compounds and expression of flavonoid biosynthesis related genes in *Achillea pachycephala* Rech.f. Phytochemistry.

[43] Hodaei M., Rahimmalek M., Arzani A., Talebi M. (2018). The effect of water stress on phytochemical accumulation, bioactive compounds and expression of key genes involved in flavonoid biosynthesis in *Chrysanthemum morifolium* L.. Ind. Crop. Prod..

[44] Nabavi S.M., Šamec D., Tomczyk M., Milella L., Russo D., Habtemariam S., Suntar I., Rastrelli L., Daglia M., Xiao J. (2020). Flavonoid biosynthetic pathways in plants: Versatile targets for metabolic engineering. Biotechnol. Adv..

[45] Saito K., Yonekura-Sakakibara K., Nakabayashi R., Higashi Y., Yamazaki M., Tohge T., Fernie A.R. (2013). The flavonoid biosynthetic pathway in Arabidopsis: Structural and genetic diversity. Plant Physiol. Biochem..

[46] Nakabayashi R., Mori T., Saito K. (2014). Alternation of flavonoid accumulation under drought stress in *Arabidopsis thaliana*. Plant Signal Behav..

[47] Bai Y.C., Li C.L., Zhang J.W., Li S.J., Luo X.P., Yao H.P., Chen H., Zhao H.X., Park S.U., Wu Q. (2014). Characterization of two tartary buckwheat R2R3-MYB transcription factors and their regulation of proanthocyanidin biosynthesis. Physiol. Plant.

[48] Savoi S., Wong D.C., Arapitsas P., Miculan M., Bucchetti B., Peterlunger E., Fait A., Mattivi F., Castellarin S.D. (2016). Transcriptome and metabolite profiling reveals that prolonged drought modulates the phenylpropanoid and terpenoid pathway in white grapes (*Vitis vinifera* L.). BMC Plant Biol..

[49] Yang L., Qiao L., Su X., Ji B., Dong C. (2022). Drought Stress Stimulates the Terpenoid Backbone and Triterpenoid Biosynthesis Pathway to Promote the Synthesis of Saikosaponin in Bupleurum chinense DC. Roots. Molecules.

[50] Dimopoulos N., Tindjau R., Wong D.C.J., Matzat T., Haslam T., Song C., Gambetta G.A., Kunst L., Castellarin S.D. (2020). Drought stress modulates cuticular wax composition of the grape berry. J. Exp. Bot..

[51] Ma D., Sun D., Wang C., Li Y., Guo T. (2014). Expression of flavonoid biosynthesis genes and accumulation of flavonoid in wheat leaves in response to drought stress. Plant Physiol. Biochem..

[52] Yang L., Zhao Y., Zhang Q., Cheng L., Han M., Ren Y., Yang L. (2019). Effects of drought-re-watering-drought on the photosynthesis physiology and secondary metabolite production of Bupleurum chinense DC. Plant Cell Rep..

[53] Bourmaud A., Gallien S., Domon B. (2016). Parallel reaction monitoring using quadrupole-Orbitrap mass spectrometer: Principle and applications. Proteomics.

