# Supplementary material for: Multi-Omics Elucidates Difference in Accumulation of Bioactive Constituents in Licorice (Glycyrrhiza uralensis) under Drought Stress
Source: Molecules. 2023 Oct 12;28(20):7042. doi: 10.3390/molecules28207042 (PMC10609028; doi:10.3390/molecules28207042)
Supplement: Supplementary file 1 [file molecules-28-07042-s001.zip › Supplementary figures_revised.pdf]

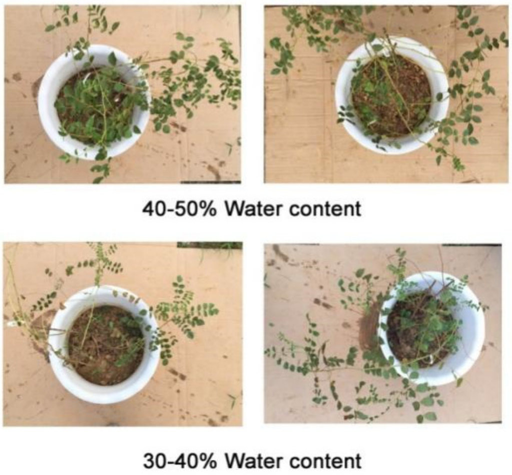

Figure S1. Morphology and phenotype profiles of four licorice seedling groups after 50 days under different water deficit conditions.

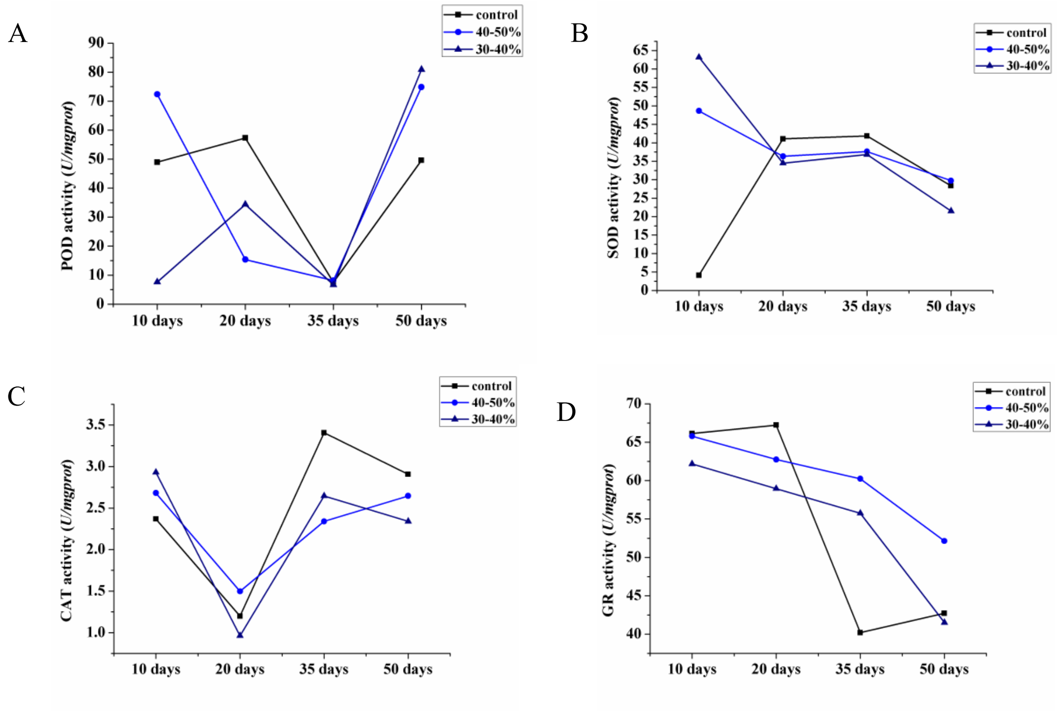

Figure S2. Dynamic contents of antioxidases POD (A), SOD (B), CAT (C), and GR (D) in all licorice groups of different drought treatments.

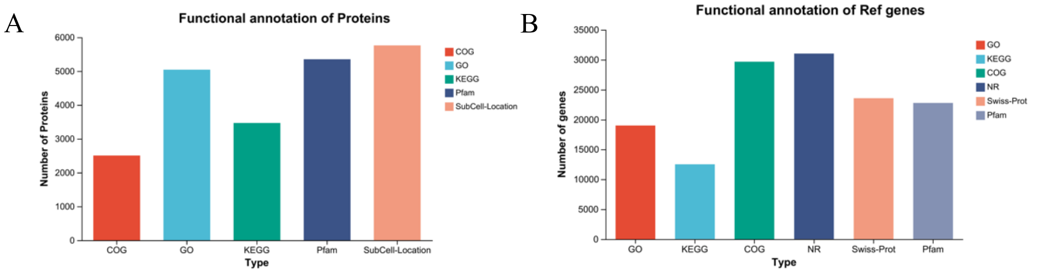

Figure S3. The functional annotation of all proteins (A) and genes (B) from Pfam, GO, KEGG, SubCell-Location and COG.

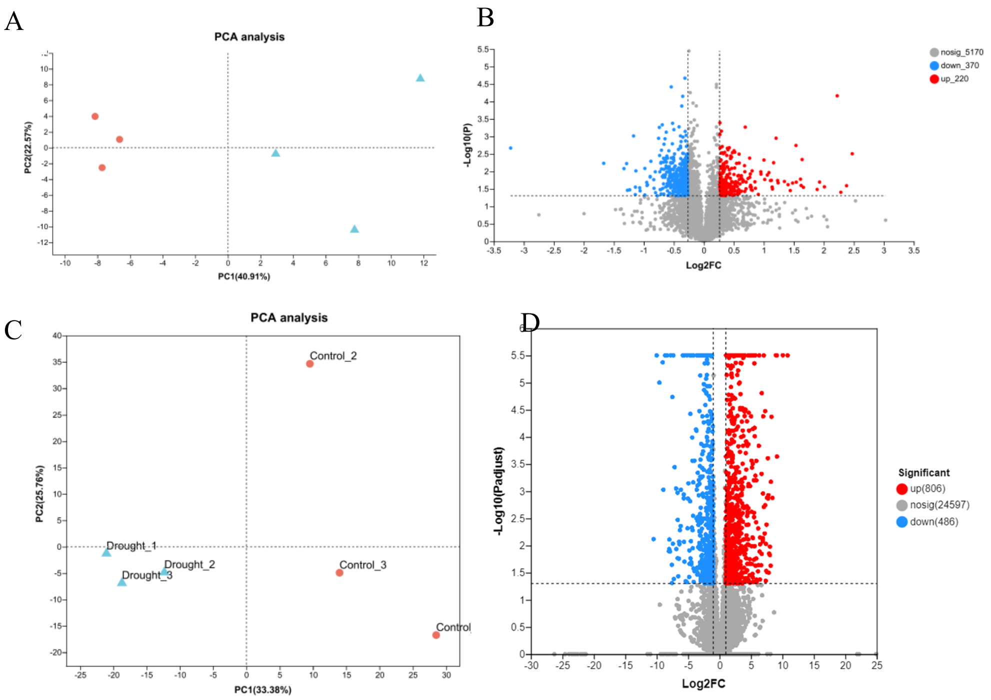

Figure S4. Principal component analysis (PCA) of DEPs (A) and DEGs (C); Volcano map analysis of DEPs (B) and DEGs (D) (red, green, and gray points represent upregulated, downregulated, and unchanged genes, respectively).

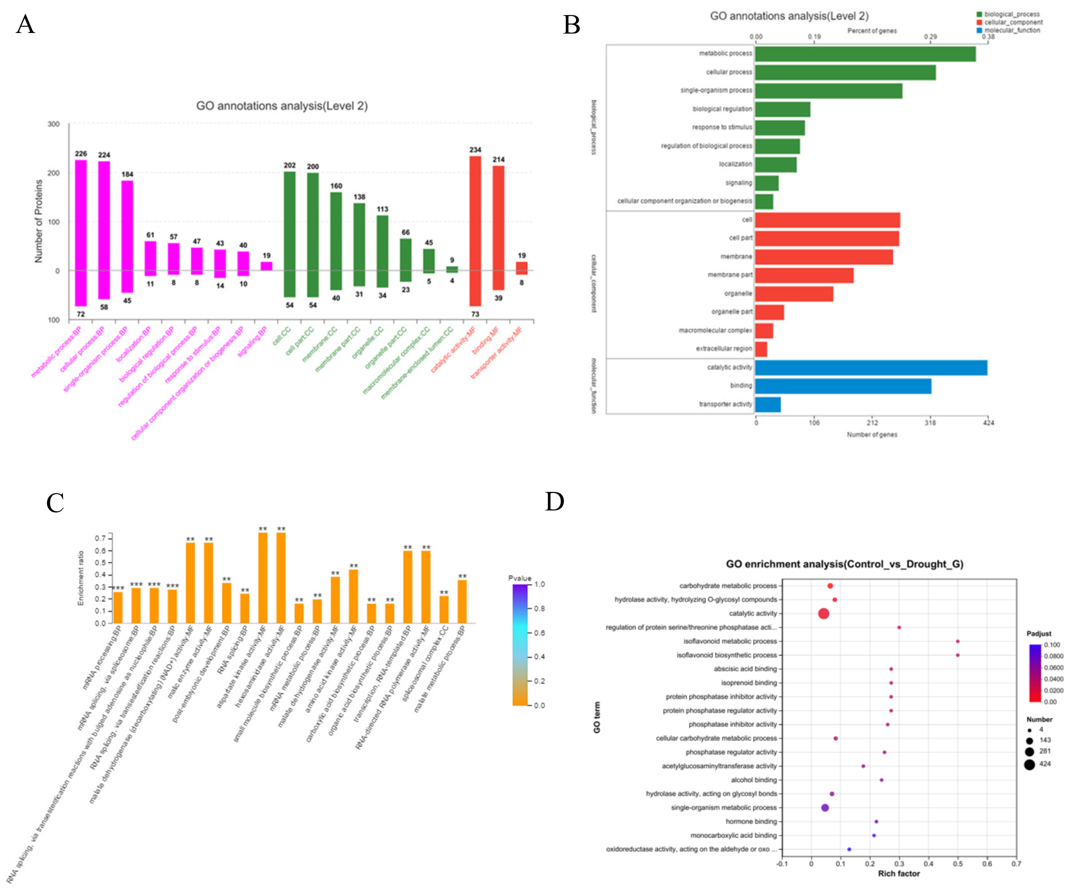

Figure S5. Gene ontology (GO, cellular component, biological process and molecular function) classification of top 20 terms of DEPs (A) and DEGs (B); GO enrichment of top 20 terms of DEPs (C) and DEGs (D); The top 20 pathways ($p \leq 0.05$) were used to generate the illustration.

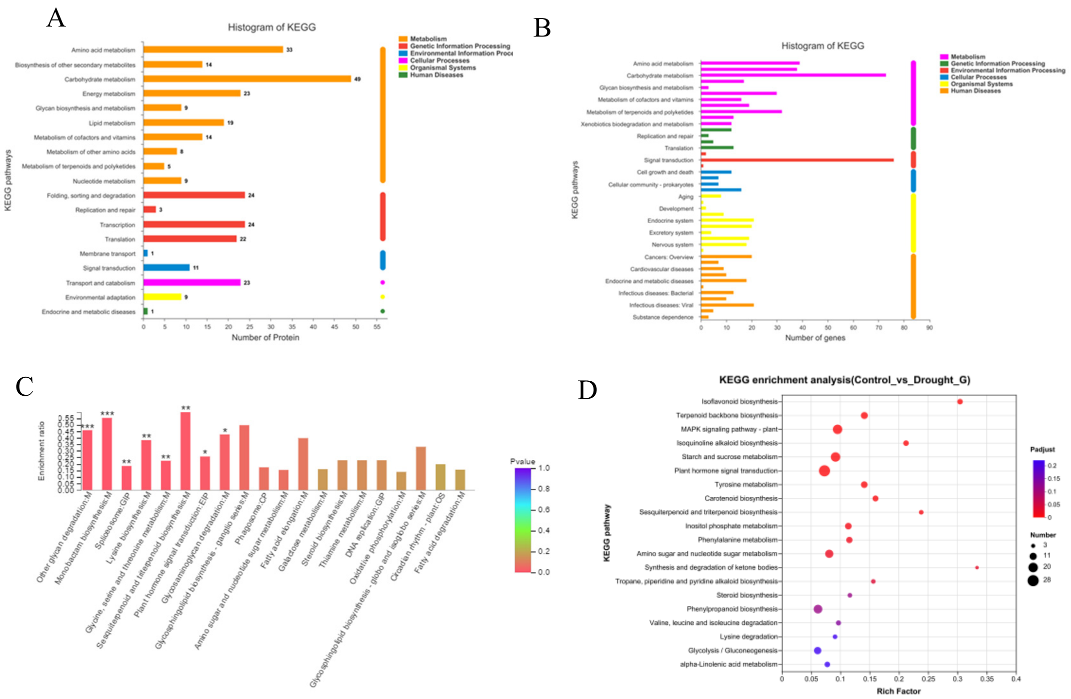

Figure S6. Kyoto Encyclopedia of Genes and Genomes (KEGG) annotation of top 20 pathways of DEPs (A) and genes (B); KEGG enrichment of top 20 pathways of DEPs (C) and DEGs (D); The top 20 pathways ($p \leq 0.05$) were used to generate the illustration.

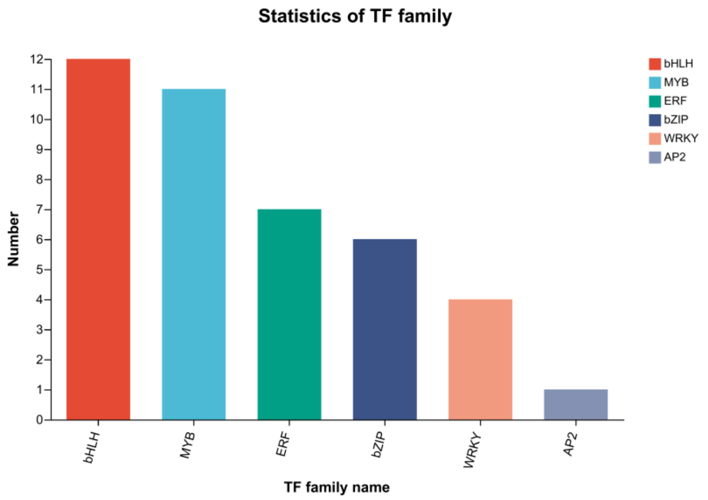

Figure S7. Transcription Factor Prediction of DEGs in licorice.

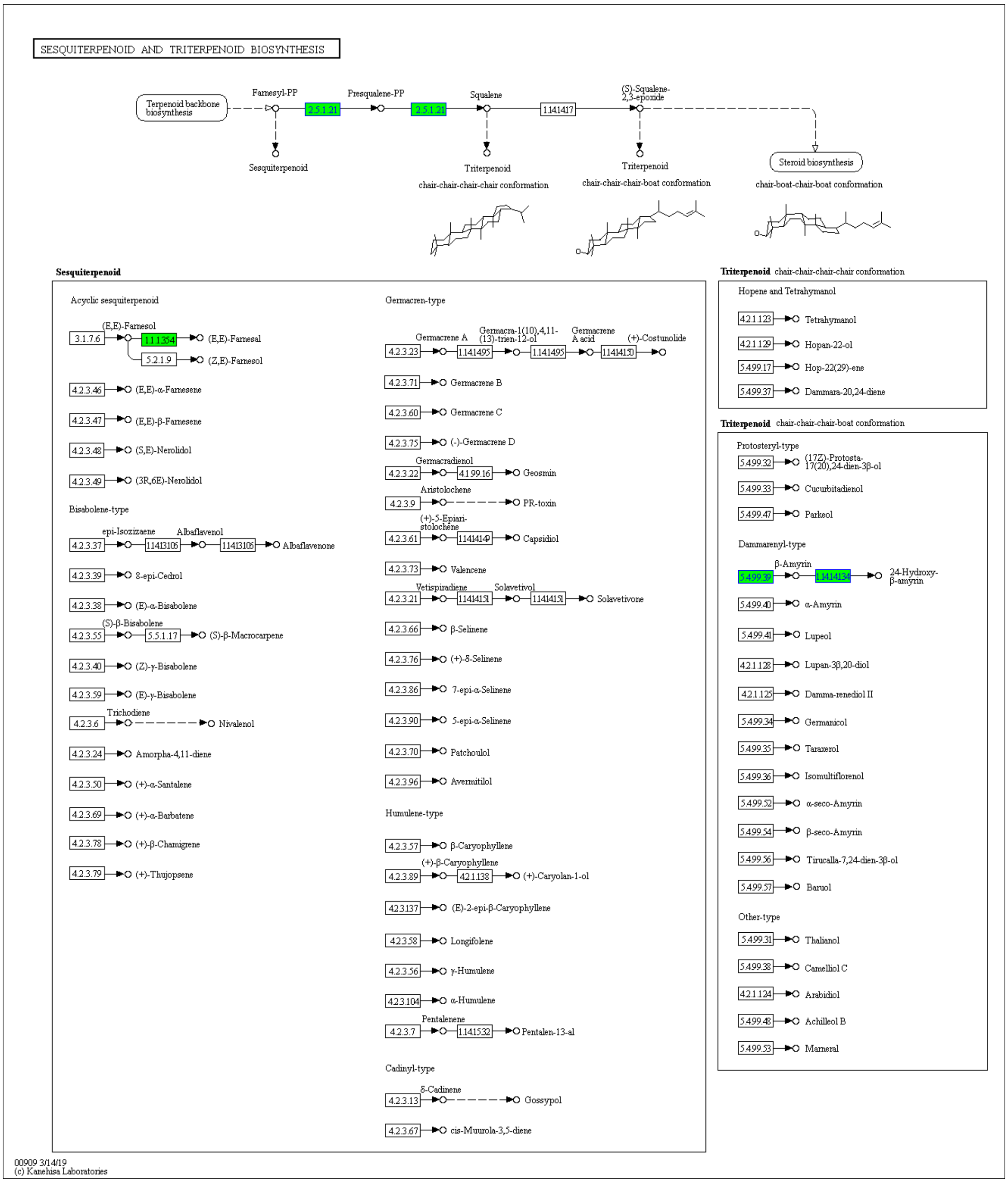

Figure S8. Metabolic pathway maps of differentially expressed proteins involved in sesquiterpenoid and triterpenoid biosynthesis in KEGG enrichment (Green for DEPs)

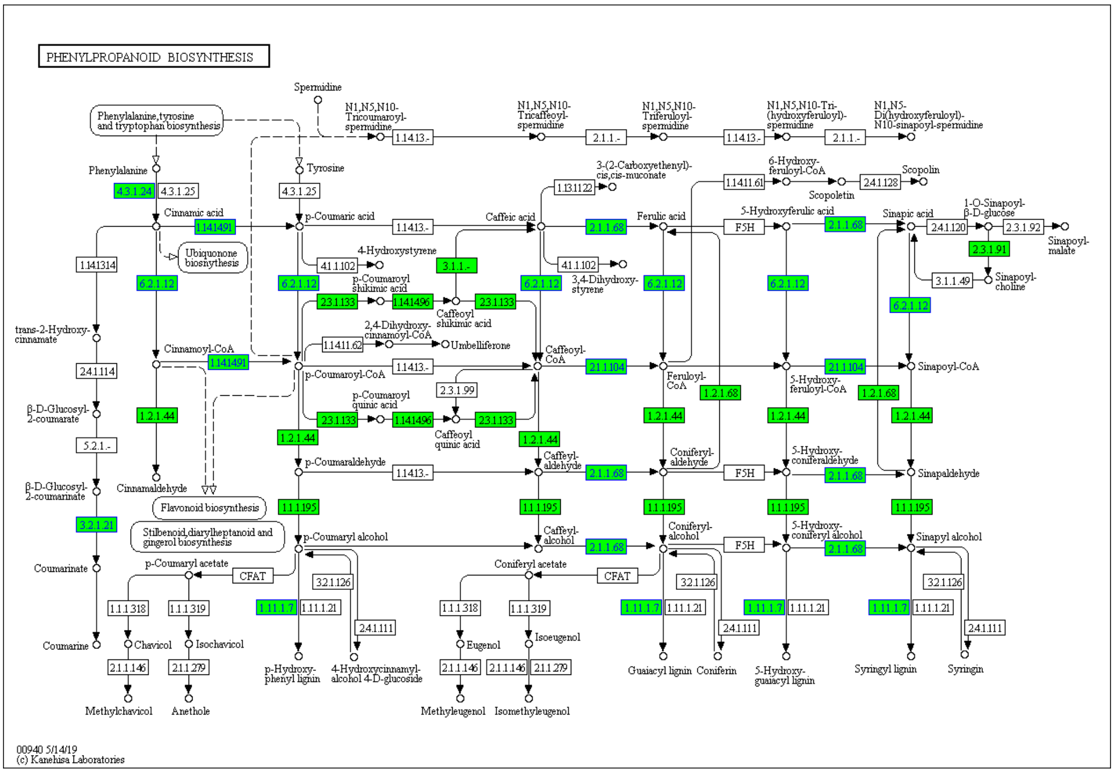

Figure S9. Metabolic pathway maps of differentially expressed proteins involved in phenylpropanoid biosynthesis in KEGG enrichment (Green for DEPs).

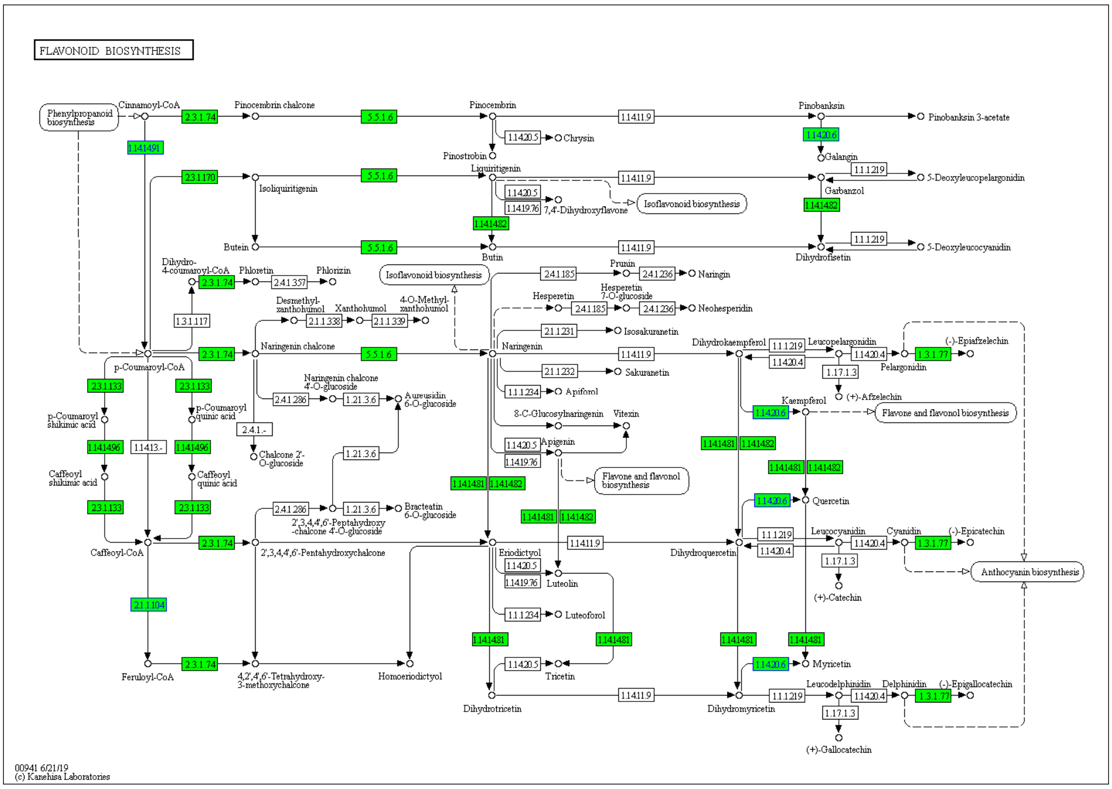

Figure S10. Metabolic pathway maps of differentially expressed proteins involved in flavonoid biosynthesis in KEGG enrichment (Green for DEPs).
